# Insecticide Effect of Zeolites on the Tomato Leafminer *Tuta absoluta* (Lepidoptera: Gelechiidae)

**DOI:** 10.3390/insects7040072

**Published:** 2016-12-02

**Authors:** Caroline De Smedt, Veerle Van Damme, Patrick De Clercq, Pieter Spanoghe

**Affiliations:** Department of Crop Protection, Faculty of Bioscience Engineering, Ghent University, Coupure Links 653, Ghent 9000, Belgium; veerle.vandamme@ugent.be (V.V.D.); patrick.declercq@ugent.be (P.D.C.); pieter.spanoghe@ugent.be (P.S.)

**Keywords:** topical, residual, oviposition, zeolites, tomato leafminer

## Abstract

(1) Background: The tomato leafminer *Tuta absoluta* (Lepidoptera: Gelechiidae) is a key tomato insect pest. At present, it is considered to be a serious threat in various countries in Europe, North Africa, and Middle East. The extensive use and the developed resistance of *T. absoluta* to spinosad causes some concern, which leads to the need for alternative products. (2) Materials and Methods: Several laboratory experiments were conducted to investigate the ovicidal properties of a zeolite particle film on *T. absoluta*. The toxicity of three different zeolites and six zeolite formulations to *T. absoluta* eggs and larvae was determined using different exposure methods. (3) Results: In general, the formulated zeolites yielded higher egg and larvae mortality values, especially when the zeolite particle film was residually applied. Notable differences in mortality rates from exposure to zeolites compared to other products, such as kaolin, its formulated product Surround, and the insecticide spinosad, were observed. Kaolin and Surround exhibited little or no effect for both application methods, while the hatch rate was reduced by 95% when spinosad was applied topically. Spinosad yielded egg and larvae mortality rates of 100% for both application methods. Additionally, increased oviposition activity was observed in adults exposed to the wettable powder (WP) formulations. These WP formulations increased egg deposition, while Surround and spinosad elicited a negative oviposition response. (4) Conclusions: It can be derived that the tested products, zeolites BEA (Beta polymorph A), FAU (Faujasite), LTA (Linde type A), and their formulations, had no real insecticidal activity against the eggs of *T. absoluta*. Nevertheless, egg exposure to zeolites seemed to affect the development process by weakening the first instar larvae and increasing their mortality. Subsequently, based on the choice test, no significant difference was observed between the number of eggs laid on the treated leaves and control leaves.

## 1. Introduction

*Tuta absoluta* (Lepidoptera, Gelechiidae), known as the South American tomato leafminer or pinworm, is one of the most devastating pests for tomato crops (*Solanum lycopersicum* L.), both in greenhouse and open-field locations in different parts of the world [1]. It originates from South America and was first described in Peru in 1917 as *Phthorimaea absoluta* (Meyrick, 1917) [2]. Recently, *T. absoluta* has also become a serious threat to tomato production in the Mediterranean region [3]. In Europe, this pest was first detected at the end of 2006 in the northern part of Castellón de la Plana in eastern Spain [4]. Since then, it has rapidly invaded other European countries and spread throughout the Mediterranean basin, including parts of North Africa and the Middle East where it immediately reached damaging levels [2,5,6].

This pest attacks leaves, flowers, stems, and fruits at any developmental stage, from seedlings to mature plants. In the absence of control strategies, yield losses can reach 80%–100% [2]. The damage is caused by the larvae mining the leaves and, sometimes, also the tomato fruits. The larvae feed on the mesophyll, which affects the photosynthetic capacity of the crop, decreases the production, and makes the tomatoes unsuitable for the market [7,8].

Nowadays, biological control, based on the predators *Nesidiocoris tenuis* (Hemiptera: Miridae) and *Macrolophus pygmaeus* (Hemiptera: Miridae), has been used to regulate the *T. absoluta* population [4]. Nevertheless, in many countries (South America, Italy, Spain, etc.) chemical control still is the main method to control *T. absoluta*. In order to decrease the damage caused by *T. absoluta*, horticultural growers applied insecticides [1,7]. They applied the chemical products more than twice a week during a single cultivation period, which not only resulted in food and environmental contamination, but also increased the cost of production and reduced the number of natural enemies of the pest [9]. Furthermore, extensive use of insecticides may lead to the development of resistance in insect populations. Development of resistance in *T. absoluta* populations was previously reported in South America [7,10,11,12]. Studies have shown that *T. absoluta* can develop resistance to many classes of insecticides when resistance management strategies are not properly established. Accordingly, high risks are involved in the use of insecticides based on spinosyns, one of the few classes of insecticides still effective against *T. absoluta* in South America [13,14].

Subsequently, the bioinsecticide “emamectin benzoate” is a new macrocyclic lactone insecticide derived from the avermectin family of natural products. These products have been developed for the control of Lepidoptera pests on a variety of vegetable crops worldwide, with a particular efficacy against *T. absoluta* [15]. Mortality rates of 90% were observed, which was similar to the results obtained by Lopez et al. [15,16].

However, Campos et al. [17] have already detected low levels of resistance of *T. absoluta* to spinosad. Notwithstanding the good results of emamectin benzoate, farmers should be careful using this product when natural enemies are involved in the control of the pest [15]. Subsequently, the introduction of the European Directive on the sustainable use of pesticides (2009/128/EC) requires that all professional users of pesticides follow the general principles of IPM (integrated pest management). Thus, the implementation of environmentally safe alternatives, reducing the use of chemicals, should contribute to the sustainability of tomato production.

One of the alternatives is the use of zeolites as an insecticide. Zeolites represent a broad range of natural or synthetic microporous, crystalline aluminosilicates. Generally, their structure is built of [SiO_4_]^4−^ and [AlO_4_]^5−^ tetrahedra, linked by the sharing of the oxygen atoms [18]. Zeolites are used for a great number of applications in different domains, including agriculture [19]. The use of particle films on plant surfaces is intended to prevent most of the negative effects that occur with the current application of chemical plant protection products. Moreover, such particle films might also provide a number of beneficial effects in terms of reduction in pesticide use, control of pests and diseases, water efficiency, increase in crop yield, and tolerance to abiotic stress [19].

In addition to the applied product, insecticide susceptibility also varies with the life stage of an insect [20]. Although the egg stage is sometimes perceived as the most vulnerable, it is a difficult target for insecticide application because the sessile condition of the eggs are often at concealed sites [21,22,23,24]. In addition, egg structure and physiology protect the developing embryos and may minimize insecticide penetration [21,24,25]. The insect chorion is a compound set of envelopes, remarkably effective in providing the oocyte with protection against possible disadvantageous environmental influences like desiccation, water loss, bacterial infection, and physical destruction. On the other hand, the egg shell enables gas exchange and maintenance of the water balance [26,27]. However, this does not apply to female insects that lay their eggs inside the leaf or that protect their eggs with scales and other materials.

This study aimed to assess the insecticidal effect of zeolites on *T. absoluta*. The treatments were mainly targeted against eggs and larvae. Ovicidal properties were studied by spraying the eggs directly (topically) or by treating leaves before oviposition (residually). A final experiment was done to assess whether the used products had repellent or attractant properties to adult females. This oviposition behavior of females was determined by choice tests.

## 2. Materials and Methods

### 2.1. Plants

Tomato seedlings (*Solanum* *lycopersicon* L. cv. Madison F1) were obtained from the company BPK Duffel N.V. (Duffel, Belgium) and placed in a greenhouse at 25 ± 1 °C, 65% ± 15% RH, and 16:8 h (lightness:darkness, L:D).

### 2.2. Insects

Eggs of *T. absoluta* were obtained from a laboratory colony maintained on tomato plants in controlled conditions (25 ± 1 °C, 65% ± 15% RH, 16:8 h (L:D)) at the Institute for Agricultural and Fisheries Research (ILVO) in Merelbeke, Belgium. This colony was started in 2011 with individuals collected in commercial fields of an organic tomato cultivation in Sint-Martens-Lennik, Belgium.

### 2.3. Insecticide Materials

Three commercially available zeolite types, selected based on the results of De Smedt et al. [28], together with two formulations of each type were compared with a commercial insecticide and a control treatment. Additionally, two biological equivalents of the zeolites, namely kaolin and its commercial formulation “Surround” (Tessenderlo Kerley, Phoenix, AZ, USA), were taken into account (Table 1).

### 2.4. Topical and Residual Bioassays

To evaluate the toxicity of zeolites against *T. absoluta* eggs and larvae hatched from these eggs, a test was designed, based on topical and residual exposure bioassays. For the topical exposure bioassays, eggs were collected from tomato leaves in the laboratory colony after 24 h of oviposition and gently transferred to tomato leaf discs. To keep the tomato leaf discs turgid for more than one week, they were deposited on top of an agar layer (1.5% concentration) in Petri dishes 35 mm in diameter. Subsequently, the test products (Table 1) were sprayed on the leaf discs with eggs in the open dishes. A fine nozzle sprayer, connected to a 100 mL Erlenmeyer, was used at a pressure of 1 bar. The residual exposure bioassays followed a similar protocol, with the exception that the eggs were placed on the tomato leaf discs 24 h after spraying. For both topical and residual bioassays, three concentrations were tested—all test products were dispersed in distilled water to obtain concentrations of 400, 4000, and 20,000 mg·L^−1^. Control treatments consisting of distilled water were also included. There were eight replicates per treatment and one replicate consisted of one leaf disc with five eggs. All treated Petri dishes were kept in controlled conditions (25 ± 1 °C, 65% ± 15% RH, 16:8 h (L:D)) until they were evaluated. Egg mortality was determined by scoring the number of unhatched eggs 9 days after treatment, and larval survival was determined by scoring the number of living larvae in the leaf discs 9 days after treatment.

### 2.5. Choice Tests for Oviposition Behavior

Oviposition site preference of *T. absoluta* female adults was studied by using a simultaneous choice arrangement. For these tests, groups of five adult females and five adult males (distinguished using morphological characteristics of pupae under a stereomicroscope) were released within a cage (30 cm × 30 cm × 30 cm) and allowed to mate [29]. After 24 h, two plants were placed inside the cage—one that had been exposed to one of the test products (Table 1) and one untreated plant. Three concentrations were tested—all test products were dispersed in distilled water to obtain concentrations of 400, 4000, and 20,000 mg·L^−1^. Control treatments (i.e., a cage containing two untreated plants) were also included. Each product was tested in four separate cages.

The experiment was completely randomized. After 24 h, the number of eggs deposited in each plant was counted to assess the oviposition preference of the *T. absoluta* females. This short time period was chosen to exclude the possibility that suitable oviposition sites would become saturated during the course of the experiment.

### 2.6. Data Analysis

Mortality data obtained from concentration–response bioassays were corrected by the mortality observed in the control treatment [30] using the formula:

$$\mathrm{Corrected}\;\mathrm{mortality}\;(\%)=\frac{P-P_{0}}{100-P_{0}}100$$

where P is the percent mortality of treated insects and P_0_ is the percent mortality of insects in the control treatment. This adjusted value is permissible when the mortality in the control treatment does not exceed 20% or when mortality data are based on a sufficiently large number of replications [31]. Normality of the data was firstly tested using the Kolmogorov-Smirnov test (*p* > 0.05). The mortality values were analyzed using a four-way analysis of variance (ANOVA) with the following factors: product, dose, life stage, and application method.

The oviposition activity of adult females was expressed by an oviposition activity index (OAI) calculated using the formula:

$$\mathrm{OAI}=\frac{N_{t}-N_{c}}{N_{t}+N_{c}}$$

where N_t_ is the number of eggs laid on plants exposed to the test solution and N_c_ is the number of eggs laid on the untreated plants [32]. The OAI ranges from −1 to +1, meaning that 0 indicates a neutral response, a negative value indicates deterrence, and a positive value indicates a stimulant effect. A Kolmogorov-Smirnov test was used to indicate the normality (*p* > 0.05). A Student’s *t*-test (*p* < 0.05) was used to test for significant differences between the number of eggs laid on the treated and the control plants.

Data analysis was performed using a statistical software program (SPSS Version 12.0, SPSS Inc., Chicago, IL, USA).

## 3. Results

### 3.1. Topical and Residual Bioassays

**Figure 1:**
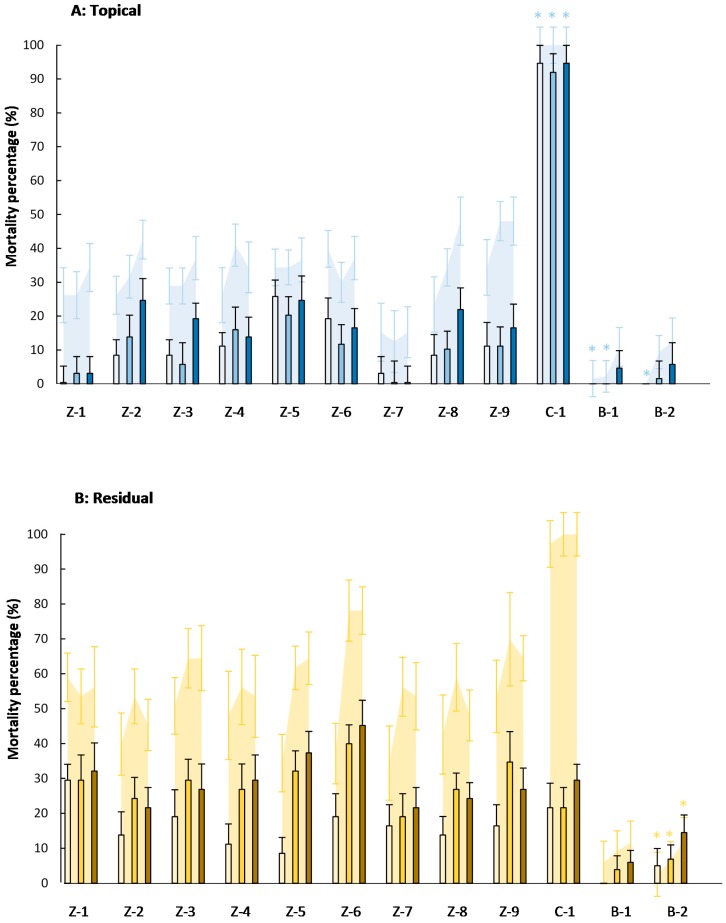
Percentage of corrected mortality (±SE) of *Tuta absoluta* eggs (bars) and eggs + larvae (area) in topical (**A**) and residual (**B**) exposure bioassays, using different concentrations of the insecticide materials (Table 1)—400 (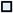, 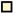), 4000 (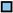, 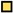), and 20,000 (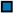, 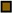) mg·L^−1^. Asterisks indicate no significant differences (*p* < 0.05) between eggs and eggs + larvae mortality (*n* = 8).

Results from bioassays testing the effects of topical and residual exposure are shown in Figure 1. The mortality registered in the control treatments was lower than 10% in all assays (Supplementary Materials, Tables S1 and S2). Therefore, the corrected mortality was used to present the results.

In general, and particularly in the topical application method, higher mortality values were noticed for the formulated zeolites as compared to the mortality values for the non-formulated zeolites. However, none of the zeolites were significantly different in mortality.

Despite no significant differences between the zeolite products, it can be deduced that the outcome of this bioassay was affected by the application method. To verify this statement, a four-way analysis of variance (ANOVA) was performed (Table 2).

No four- or three-factorial interactions were observed among the factors dose, application method, product, and insect life stage for the mortality of eggs and eggs + larvae. However, significant interactions (*p* < 0.05) were obtained for application method × product and application method × life stage, which indicates that the product and the life stage influenced the effect of the application method on the mortality of *T. absoluta*. The main factors application method (*p* < 0.01) and life stage (*p* < 0.001) also had a significant impact on the mortality.

Notwithstanding the difference between the application methods, little biological effect on eggs was observed when a zeolite particle film was sprayed topically or residually onto leaves. Although *T. absoluta* egg viability was little affected by the zeolites, the mortality of neonate larvae hatching on treated leaves was significantly higher for all zeolites. Larval mortality increased sharply to 60%, indicating that the zeolites have larvicidal properties. These larval survival results were also significantly different from the control.

Nevertheless, differences of the mortality rates were observed when using zeolites compared to the alternative products. First of all, kaolin had very little or no larvicidal effect compared to the zeolites. The opposite effect was observed when using the commercial formulation of spinosad. In both exposure treatments, it was observed that almost all of the larvae that hatched out of egg masses treated with spinosad died within 48 h. Spinosad was the least active after the residually application at all concentrations tested, yielding 21.64%–29.48% mortality for concentrations ranging between 400 and 20,000 mg·L^−1^. Figure 1 clearly points out the significant differences between the zeolites and their formulations regarding the chemical plant protection product.

### 3.2. Choice Tests for Oviposition Behavior

**Figure 2:**
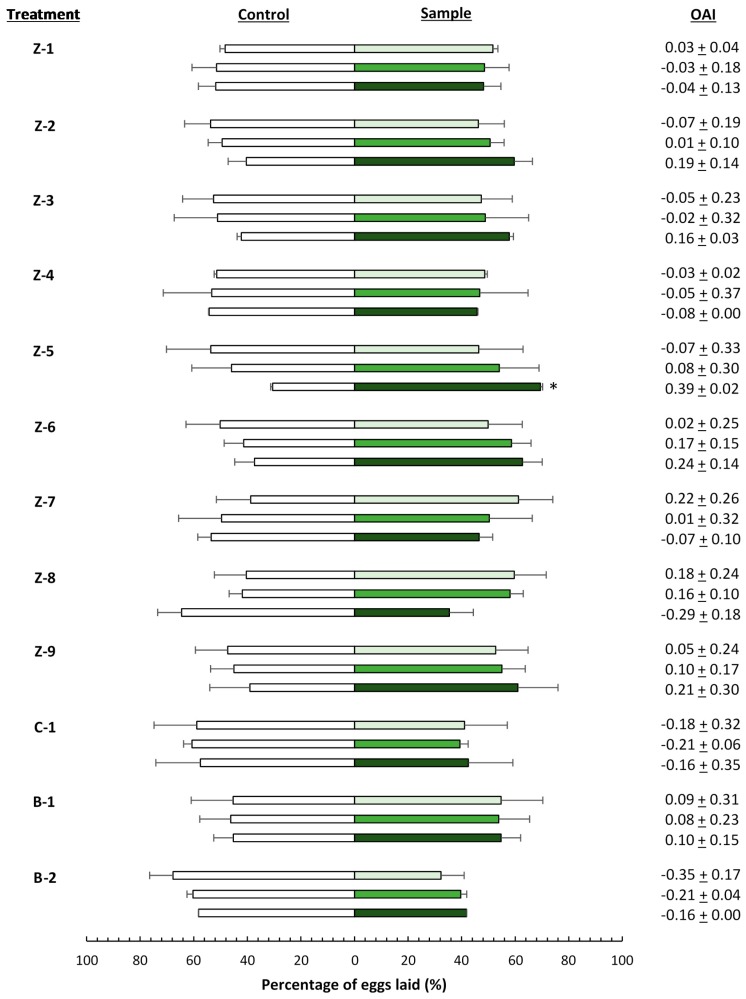
Oviposition response of *Tuta absoluta* to plants treated with different concentrations of the insecticide materials (Table 1). White bars indicate the mean percentage of eggs laid on control leaves, green bars indicate the mean percentage of eggs laid on leaves treated with a concentration of 400 (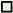), 4000 (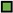), and 20,000 (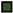) mg·L^−1^. Asterisks indicate significant differences (*p* < 0.05) in oviposition activity between treated and control leaves (*n* = 4). OAI: oviposition activity index.

The results of the oviposition bioassays are illustrated in Figure 2. The number of eggs laid by *T. absoluta* females on the treated leaves did not differ significantly from the control, with the exception of leaves treated with 20,000 mg·L^−1^ of zeolite 5.

## 4. Discussion

### 4.1. Topical and Residual Toxicity

Topical and residual toxicity tests were performed on the eggs of *Tuta absoluta* in order to examine whether zeolites could have a desiccating effect on eggs or not. In order to fight the tomato leafminer, it is of great importance to treat their eggs. Although the chorion surface layer has limited permeability to ovicidal and toxic substances, some chemicals can pass through it. These compounds can adversely affect embryonic development or cause death [33,34,35,36,37]. In particular, before egg hatching, egg exposure to zeolites seemed to significantly affect the development process by weakening the first instar larvae and increasing their mortality. Exposure of larvae to insecticidal compounds occurs through direct body contact as well as ingestion of residues on leaf surfaces [38]. There should be no difference between the numbers of surviving larvae for both application methods because the larvae were not treated after hatching.

Based on the Si/Al composition, described by De Smedt et al. [28], zeolite 1 (Si/Al: 11.84) and zeolite 4 (Si/Al: 15.40) can both be classified as hydrophobic zeolites, while zeolite 7 (Si/Al: 1.15) can be classified as hydrophilic. It was noticed that the ovicidal effect of the tested zeolites correlated well with their hydrophobic properties. This is in contrast with the phenomenon observed by Hoffmann et al. [39]. They noticed a correlation between the ovicidal activity of neonicotinoids against the plum curculio *Conotrachelus nenuphar* (Coleoptera: Curculionidae) and the octanol-water partitioning coefficient (log K_ow_) of these compounds. Hydrophilic compounds are unlikely to reach target sites within the embryo, since the lipid layers of the insect chorion provide a general barrier to hydrophilic materials [39]. However, the results of the adsorption experiment, described by De Smedt et al. [28], indicated that hydrophobic zeolites preferentially adsorb the intermediate and nonpolar PPPs. Therefore, it can be presumed that the more hydrophobic zeolites are more attached to the hydrophobic egg surface and are more able to desiccate the eggs and the leaf surface. Low relative humidity can prevent embryo development and egg hatching [40]. Norhisham et al. [41] found that dehydration of the *Dinoderus minutus* Fabricius (Coleoptera: Bostrychidae) egg leads to contraction and shrinkage of the chorion and the embryo. This effect on eggs caused by loss of water has also been reported by Woods and Singer [42] on Lepidoptera. Eggs of Lepidoptera in general have no special morphological or physiological equipment for water uptake, although the eggshell of some species may be more porous over its ventral surfaces than on lateral or upper surfaces [42].

The lower hatching rates of the *T. absoluta* eggs for the topical application were rather unexpected. A decrease in adhesion of the insect eggs on the leaf surface can be an explanation for the higher mortality in the residual trials. Adhesive fluids have been repeatedly reported to surround insect eggs and glue them to substrates [43,44]. The eggs of many lepidopteran species are tightly glued onto the lower leaf surface [45].

Plants can also have an impact on the mortality, which explains the higher mortality for the residual bioassay. The eggs of herbivorous insects are usually closely associated with leaf surfaces. As a consequence of living in these microclimates modified by their host plants, these eggs may take advantage of the leaf physiology [46]. However, eggs laid on a leaf are enclosed by the leaf’s boundary layer, consisting of leaf volatiles and atmospheric gases, which may affect embryonic development [47]. A plant may respond to insect eggs by forming necrotic tissue at the site of egg deposition, where humidity decreases and local temperature increases [48,49,50]. As a result, the egg will probably desiccate and the stressed embryo will die [47].

Subsequently, it is possible that larvae hatch at the side of the leaf surface and directly penetrate into the leaf. The probability that the zeolite particles and newly emerged larvae come into contact with each other increases for the residual method.

In addition to the zeolites, other products were also tested, such as kaolin, Surround, and spinosad. Kaolin and Surround are products that control insects by creating a particle barrier on plant surfaces that irritates and repels them, rather than actually killing these insects [51]. Despite the low effect on hatching rate, there appeared to be a slight tendency toward reduced hatching when eggs were laid onto kaolin residues. Similar results were also observed in other studies [52,53,54].

As for spinosad, the obtained results were in line with those obtained by Temerak [55] and Hanan and Samya [56], who found that spinosad produced 100% mortality of the egg masses of *Spodoptera littoralis* (Lepidoptera: Noctuidae) after hatching. Spinosad was the least active in its indirect ovicidal activity at all concentrations tested by Hanan and Samya [56], recording 0%–45% inhibition for concentrations ranging between 0.1 and 100 mg·L^−1^. However, the high toxicity of spinosad on lepidopterous eggs was also supported by Boiteau and Noronha [57], who found that spinosad residues caused high immediate (24 h after exposure) contact mortality in the European corn borer, *Ostrinia nubilalis* (Lepidoptera: Crambidae). Dagli and Bahsi [38] also noticed that topical exposure of *Orius majusculus* (Hemiptera: Anthocoridae) adults to spinosad resulted in greater mortality compared to the mortalities during residue tests. These findings can be explained by the fact that spinosad is effective on target insects through both ingestion and contact exposure. It is more a potent larvicide, which is in line with our findings of 100% larval mortality. Nevertheless, it can also have an ovicidal effect, mostly when it is mixed into organic solvents [57]. It is not highly systemic, but does possess some leaf-penetrating characteristics, which clarifies the high egg mortality after topical exposure [58]. This indicates that spinosad can penetrate the egg, causing the exposed egg to stop further development.

### 4.2. Choice Tests for Oviposition Behavior

Zeolites work by creating a barrier film by covering the leaves with a white powdery film, which adheres and irritates insects. Additionally, using zeolites on plants can help to repel many types of insects. Identification of suitable oviposition sites is a critical feature of insects’ life history because it ultimately influences the survivorship of their progeny [59].

Oviposition behavior is influenced by visual, tactile, and olfactory cues, with the first considered to be of primary importance when zeolites are used [60]. The layer of particle film covering the leaves reduces the attractiveness of visual cues and prevents insects from recognizing and finding plant parts on which they prefer to lay eggs [19]. Subsequently, odor of plants, the plant surface, and the plant’s interior guide egg-laying herbivorous insect females to their host plants and influence the choice of oviposition sites [61]. To be effective, complete coverage of the plant is necessary. Hence, high levels of zeolite coverage over the leaves are needed to achieve any sort of control [62]. In this study, no significant difference was observed between the number of eggs laid on the treated leaves and the control leaves. Despite these results, a slight trend was noticed in the oviposition activity of *T. absoluta* females exposed to leaves treated with the wettable powder formulations. However, this effect could be the result of the additives used in the formulations. Subsequently, olfaction also might have played a role in the increased amount of eggs laid onto the treated leaves. The effect of additives is possible here as well, given that some additives are responsible for a mild attractiveness to female insects.

The negative oviposition response towards spinosad and Surround can be clarified by the statements given above.

Nevertheless, all these findings did not significantly differ from each other. Therefore, it can be concluded that these products had no effect on the oviposition behavior of *T. absoluta*. Even kaolin and its formulated product Surround, which is already commercially used in agriculture and has insect repellent properties, did not show repellent effects on *T. absoluta*.

## 5. Conclusions

Based on the results, it can be derived that the tested products, BEA, FAU, LTA and their formulations, had no real insecticidal activity against the eggs of *T. absoluta*. Nevertheless, egg exposure to zeolites seemed to affect the development process by weakening the first instar larvae and increasing their mortality. Consequently, zeolites can be applied as a preventive control measure, but cannot control a pest that is already established. To be effective, a continuous coverage of plant material with a zeolite particle film is needed. This requires multiple applications and a better coverage than that obtained in these tests, as newly expanding foliage needs to be covered as well. However, particle films are less effective in suppressing *T. absoluta* compared with spinosad. Nevertheless, they may be an important supplement to other biological and chemical control measures in future *T. absoluta* management strategies.

Subsequently, based on the choice test, no significant difference was observed between the number of eggs laid on the treated leaves and control leaves.

## Figures and Tables

**Table 1:** Insecticide materials used in this study.

Treatment	Material Name	Formulation Type	Manufacturers
Z-1	BEA (Beta polymorph A)	Technical product	Clariant
Z-2	BEA 850 ^a^	WP ^c^	Fitofarmacia
Z-3	BEA 950 ^b^	WP	Fitofarmacia
Z-4	FAU (Faujasite)	Technical product	Zeolyst
Z-5	FAU 850 ^a^	WP	Fitofarmacia
Z-6	FAU 920 ^b^	WP	Fitofarmacia
Z-7	LTA (Linde type A)	Technical product	FMC
Z-8	LTA 800	SC ^c^	Fitofarmacia
Z-9	LTA 850	WP	Fitofarmacia
C-1	Spinosad (Conserve Pro)	SC	Dow Agrosciences B.V.
B-1	Kaolin	Technical product	Sigma Aldrich
B-2	Kaolin (Surround)	WP	Tessenderlo Group

^a,b^ The formulations with a similar letter contain the same adjuvants.; ^c^ WP: wettable powder, SC: suspension concentrate.

**Table 2:** Four-way ANOVA results indicating the effect of dose, application method, product, and life stage on the mortality of *Tuta absoluta*.

Factor	*F*	df	*p*
Dose	3.234	1	0.074
Application method	6.925	1	0.010 ^b^
Product	1.057	1	0.306
Life stage	45.472	1	0.000 ^c^
Dose × application method	0.043	1	0.836
Dose × product	0.001	1	0.971
Dose × life stage	0.004	1	0.947
Application method × product	4.883	1	0.029 ^a^
Application method × life stage	3.938	1	0.049 ^a^
Product × life stage	0.772	1	0.381
Dose × application method × product	0.000	1	0.996
Dose × application method × life stage	0.043	1	0.836
Dose × product × life stage	0.003	1	0.958
Application method × product × life stage	0.144	1	0.705
Dose × application method × product × life stage	0.000	1	0.989
Error		128	

^a,b,c^ Significant differences, with a: *p* < 0.05, b: *p* < 0.01 and c: *p* < 0.001.

## Supplementary Materials

The supplementary materials are available online at http://www.mdpi.com/2075-4450/7/4/72/s1, Table S1: Percentage corrected mortality (±SE) of Tuta absoluta eggs in topical and residual bioassays, using the insecticide materials presented in Table 1 (*n* = 8), Table S2: Percentage corrected mortality (±SE) of *Tuta absoluta* eggs and larvae in topical and residual bioassays, using the insecticide materials presented in Table 1 (*n* = 8).
