# Supplementary material for: Insecticide Effect of Zeolites on the Tomato Leafminer Tuta absoluta (Lepidoptera: Gelechiidae)
Source: Insects. 2016 Dec 2;7(4):72. doi: 10.3390/insects7040072 (PMC5198220; doi:10.3390/insects7040072)
Supplement: Supplementary file 1 [file insects-07-00072-s001.pdf]

Supplementary Materials: Insecticide Effect of Zeolites on the Tomato Leafminer *Tuta absoluta* (Lepidoptera: Gelechiidae)

Caroline De Smedt *, Veerle Van Damme, Patrick De Clercq and Pieter Spanoghe

Table S1. Percentage corrected mortality (±SE) of *Tuta absoluta* eggs in topical and residual bioassays, using the insecticide materials presented in Table 1 (n = 8).

Treatment	Egg Mortality (%)					
	Mortality			Corrected Mortality		
	400 mg·L^{-1}	4000 mg·L^{-1}	20,000 mg·L^{-1}	400 mg·L^{-1}	4000 mg·L^{-1}	20,000 mg·L^{-1}
			**Topical Bioassay**			
**Control**	7.1 ± 2.66	7.1 ± 2.66	7.1 ± 2.66			
**Z-1**	7.5 ± 3.65	10.0 ± 3.78	10.0 ± 3.78	0.4 ± 4.87	3.1 ± 4.98	3.1 ± 4.98
**Z-2**	15.0 ± 3.27	20.0 ± 5.35	30.0 ± 5.35	8.5 ± 4.55	13.9 ± 6.44	24.6 ± 6.47
**Z-3**	15.0 ± 3.27	12.5 ± 5.26	25.0 ± 3.27	8.5 ± 4.55	5.8 ± 6.35	19.2 ± 4.57
**Z-4**	17.5 ± 2.50	22.0 ± 5.54	20.0 ± 4.71	11.2 ± 3.94	16.0 ± 6.63	13.9 ± 5.84
**Z-5**	31.1 ± 3.51	26.0 ± 4.27	30.0 ± 6.15	25.8 ± 4.80	20.3 ± 5.45	24.6 ± 7.25
**Z-6**	25.0 ± 5.00	18.0 ± 4.67	22.5 ± 4.53	19.2 ± 6.12	11.7 ± 5.79	16.5 ± 5.68
**Z-7**	10.0 ± 3.78	7.5 ± 5.26	7.5 ± 3.66	3.1 ± 4.98	0.4 ± 6.35	0.4 ± 4.87
**Z-8**	15.0 ± 5.00	16.7 ± 4.14	27.5 ± 5.26	8.5 ± 6.10	10.3 ± 5.31	21.9 ± 6.38
**Z-9**	17.5 ± 5.90	17.5 ± 4.53	22.5 ± 5.90	11.2 ± 6.98	11.2 ± 5.67	16.6 ± 6.99
**C-1**	95.0 ± 3.27	92.5 ± 3.66	95.0 ± 3.27	94.6 ± 5.29	91.9 ± 5.54	94.6 ± 5.29
**B-1**	4.3 ± 2.28	4.7 ± 2.12	11.4 ± 4.04	0.0 ± 0.00	0.0 ± 0.00	4.6 ± 5.21
**B-2**	2.9 ± 2.86	8.6 ± 4.04	12.5 ± 5.26	0.0 ± 0.00	1.5 ± 5.21	5.8 ± 6.35
			**Residual Bioassay**			
**Control**	4.3 ± 2.28	4.3 ± 2.28	4.3 ± 2.28			
**Z-1**	32.5 ± 3.66	32.5 ± 6.48	35.0 ± 7.32	29.5 ± 4.56	29.5 ± 7.21	32.1 ± 8.04
**Z-2**	17.5 ± 5.90	27.5 ± 5.26	25.0 ± 5.00	13.8 ± 6.62	24.3 ± 6.02	21.6 ± 5.76
**Z-3**	22.5 ± 7.01	32.5 ± 5.26	30.0 ± 6.55	19.0 ± 7.71	29.5 ± 6.03	26.9 ± 7.27
**Z-4**	15.0 ± 5.00	30.0 ± 6.55	32.5 ± 6.48	11.2 ± 5.75	26.9 ± 7.27	29.5 ± 7.21
**Z-5**	12.5 ± 3.66	35.0 ± 5.00	40.0 ± 5.35	8.6 ± 4.51	32.1 ± 5.79	37.3 ± 6.13
**Z-6**	22.5 ± 5.90	42.5 ± 4.53	47.5 ± 6.48	19.0 ± 6.62	39.9 ± 5.38	45.2 ± 7.25
**Z-7**	20.0 ± 5.35	22.5 ± 5.90	25.0 ± 5.00	16.4 ± 6.08	19.0 ± 6.62	21.6 ± 5.76
**Z-8**	17.5 ± 4.53	30.0 ± 3.78	27.5 ± 3.66	13.8 ± 5.31	26.9 ± 4.65	24.3 ± 4.54
**Z-9**	20.0 ± 5.35	37.5 ± 7.96	30.0 ± 5.35	16.4 ± 6.08	34.7 ± 8.69	26.9 ± 6.10
**C-1**	25.0 ± 6.27	25.0 ± 5.00	32.5 ± 3.66	21.6 ± 6.99	21.6 ± 5.76	29.5 ± 4.56
**B-1**	4.0 ± 1.84	8.0 ± 3.04	10.0 ± 2.41	0.0 ± 0.00	3.9 ± 3.97	6.0 ± 3.46
**B-2**	9.1 ± 4.15	10.9 ± 3.15	18.2 ± 4.23	5.0 ± 4.94	6.9 ± 4.06	14.5 ± 5.03

Table S2. Percentage corrected mortality (±SE) of *Tuta absoluta* eggs and larvae in topical and residual bioassays, using the insecticide materials presented in Table 1 (n = 8).

Treatment	Egg + Larvae Mortality (%)					
	Mortality			Corrected Mortality		
	400 mg·L^{-1}	4000 mg·L^{-1}	20,000 mg·L^{-1}	400 mg·L^{-1}	4000 mg·L^{-1}	20,000 mg·L^{-1}
**Topical Bioassay**						
**Control**	8.6 ± 3.45	8.6 ± 3.45	8.6 ± 3.45			
**Z-1**	32.5 ± 6.48	32.5 ± 5.26	40.0 ± 5.35	26.2 ± 8.09	26.2 ± 6.95	34.4 ± 7.08
**Z-2**	32.5 ± 3.66	37.5 ± 4.53	47.5 ± 3.66	26.2 ± 5.59	31.6 ± 6.35	42.6 ± 5.73
**Z-3**	35.0 ± 3.27	35.0 ± 3.27	42.5 ± 4.53	28.9 ± 5.32	28.9 ± 5.32	37.1 ± 6.39
**Z-4**	32.5 ± 6.48	46.0 ± 4.27	40.0 ± 5.77	26.2 ± 8.09	40.9 ± 6.20	34.4 ± 7.47
**Z-5**	40.0 ± 3.33	40.0 ± 2.98	42.0 ± 4.67	34.4 ± 5.41	34.4 ± 5.16	36.6 ± 6.50
**Z-6**	45.0 ± 3.27	36.0 ± 4.00	42.5 ± 4.53	39.8 ± 5.42	30.0 ± 5.89	37.1 ± 6.39
**Z-7**	22.5 ± 7.01	20.0 ± 7.56	22.5 ± 5.90	15.2 ± 8.56	12.5 ± 9.10	15.2 ± 7.50
**Z-8**	30.0 ± 6.55	40.0 ± 3.48	52.5 ± 5.26	23.4 ± 8.14	34.4 ± 5.52	48.1 ± 7.12
**Z-9**	40.0 ± 6.55	52.5 ± 3.66	52.5 ± 5.26	34.4 ± 8.20	48.1 ± 5.80	48.1 ± 7.12
**C-1**	100.0 ± 0.00	100.0 ± 0.00	100.0 ± 0.00	100.0 ± 5.34	100.0 ± 5.34	100.0 ± 5.34
**B-1**	10.0 ± 3.48	10.6 ± 2.50	18.6 ± 3.90	1.6 ± 5.36	2.2 ± 4.66	10.9 ± 5.71
**B-2**	2.9 ± 2.86	17.1 ± 2.86	20.0 ± 5.35	0.0 ± 0.00	9.4 ± 4.92	12.5 ± 6.98
**Residual Bioassay**						
**Control**	8.6 ± 4.04	8.6 ± 4.04	8.6 ± 4.04			
**Z-1**	62.5 ± 4.27	57.5 ± 5.56	60.0 ± 9.43	59.0 ± 6.94	53.5 ± 7.88	56.3 ± 11.49
**Z-2**	45.0 ± 6.90	57.5 ± 5.56	50.0 ± 5.04	39.8 ± 8.92	53.5 ± 7.88	45.3 ± 7.34
**Z-3**	55.0 ± 5.91	67.5 ± 6.11	67.5 ± 7.07	50.8 ± 8.15	64.5 ± 8.50	64.5 ± 9.35
**Z-4**	52.5 ± 10.65	60.0 ± 8.73	57.5 ± 9.72	48.1 ± 12.64	56.3 ± 10.81	53.5 ± 11.75
**Z-5**	40.0 ± 6.17	65.0 ± 3.09	67.5 ± 4.96	34.4 ± 8.21	61.7 ± 6.19	64.5 ± 7.55
**Z-6**	42.5 ± 6.61	80.0 ± 6.17	80.0 ± 3.56	37.1 ± 8.63	78.1 ± 8.78	78.1 ± 6.83
**Z-7**	40.0 ± 8.73	60.0 ± 6.17	57.5 ± 7.51	34.4 ± 10.63	56.3 ± 8.44	53.5 ± 9.62
**Z-8**	47.5 ± 9.39	62.5 ± 7.51	52.5 ± 4.96	42.6 ± 11.33	59.0 ± 9.68	48.1 ± 7.31
**Z-9**	57.5 ± 8.31	72.5 ± 11.23	67.5 ± 3.45	53.5 ± 10.38	69.9 ± 13.42	64.5 ± 6.47
**C-1**	97.5 ± 2.36	100.0 ± 0.00	100.0 ± 0.00	97.3 ± 6.68	100.0 ± 6.25	100.0 ± 6.25
**B-1**	14.0 ± 3.87	17.0 ± 3.33	19.2 ± 3.90	5.9 ± 6.12	9.2 ± 5.74	11.6 ± 6.16
**B-2**	10.9 ± 4.15	14.6 ± 2.82	20.0 ± 4.02	2.6 ± 6.33	6.5 ± 5.39	12.5 ± 6.26

© 2016 by the authors. Submitted for possible open access publication under the terms and conditions of the Creative Commons Attribution (CC-BY) license (http://creativecommons.org/licenses/by/4.0/).
